# Pathophysiological Mechanisms and Potential Therapeutic Targets in Intracerebral Hemorrhage

**DOI:** 10.3389/fphar.2019.01079

**Published:** 2019-09-19

**Authors:** Zhiwei Shao, Sheng Tu, Anwen Shao

**Affiliations:** ^1^Department of Hepatobiliary and Pancreatic Surgery, Department of Surgery, Second Affiliated Hospital, School of Medicine, Zhejiang University, Hangzhou, China; ^2^Department of Infectious Diseases, Collaborative Innovation Center for Diagnosis and Treatment of Infectious Diseases, The First Affiliated Hospital, School of Medicine, Zhejiang University, Hangzhou, China; ^3^Department of Neurosurgery, Second Affiliated Hospital, School of Medicine, Zhejiang University, Hangzhou, China

**Keywords:** intracerebral hemorrhage, inflammation, oxidative stress, Nrf2, iron, thrombin

## Abstract

Intracerebral hemorrhage (ICH) is a subtype of hemorrhagic stroke with high mortality and morbidity. The resulting hematoma within brain parenchyma induces a series of adverse events causing primary and secondary brain injury. The mechanism of injury after ICH is very complicated and has not yet been illuminated. This review discusses some key pathophysiology mechanisms in ICH such as oxidative stress (OS), inflammation, iron toxicity, and thrombin formation. The corresponding therapeutic targets and therapeutic strategies are also reviewed.

## Introduction

Hemorrhagic stroke, including intracerebral hemorrhage (ICH) and subarachnoid hemorrhage (SAH), is associated with high mortality and morbidity (Keep et al., 2012). Despite significant progress in clinical treatment, the incidence of hemorrhagic stroke has not declined (van Asch et al., 2010). At present, although hematoma can be removed surgically to relieve the mechanical compression of peripheral brain tissue, there are still no efficient treatments for secondary brain injuries (SBIs) such as oxidative stress (OS), inflammatory response, neuronal apoptosis, and thrombin formation caused by hemorrhage (Hu et al., 2016; Zheng et al., 2016; Zhang et al., 2017; Zeng et al., 2018). These mechanisms are related to each other, but they have not been fully illustrated. Obviously, a multi-targeted neuroprotective compound would be a promising strategy to alleviate brain injury after ICH. In this review, we aim to summarize the essential mechanisms and pathological progress after ICH and to comment on potential therapeutic targets.

## Primary Injury

The initial pathological damage of cerebral hemorrhage to brain is the mechanical compression caused by hematoma. The hematoma mass can increase intracranial pressure, compressing brain and thereby potentially affecting blood flow, and subsequently leading to brain hernia (Keep et al., 2012). Subsequently, brain hernia and brain edema cause secondary injury, which may be associated with poor outcome and mortality in ICH patients (Yang et al., 2016). Unfortunately, the common treatment of brain edema (steroids, mannitol, glycerol, and hyperventilation) cannot effectively reduce intracranial pressure or prevent secondary brain injury (Cordonnier et al., 2018).

## Secondary Brain Injury

### Inflammation

**Figure 1:**
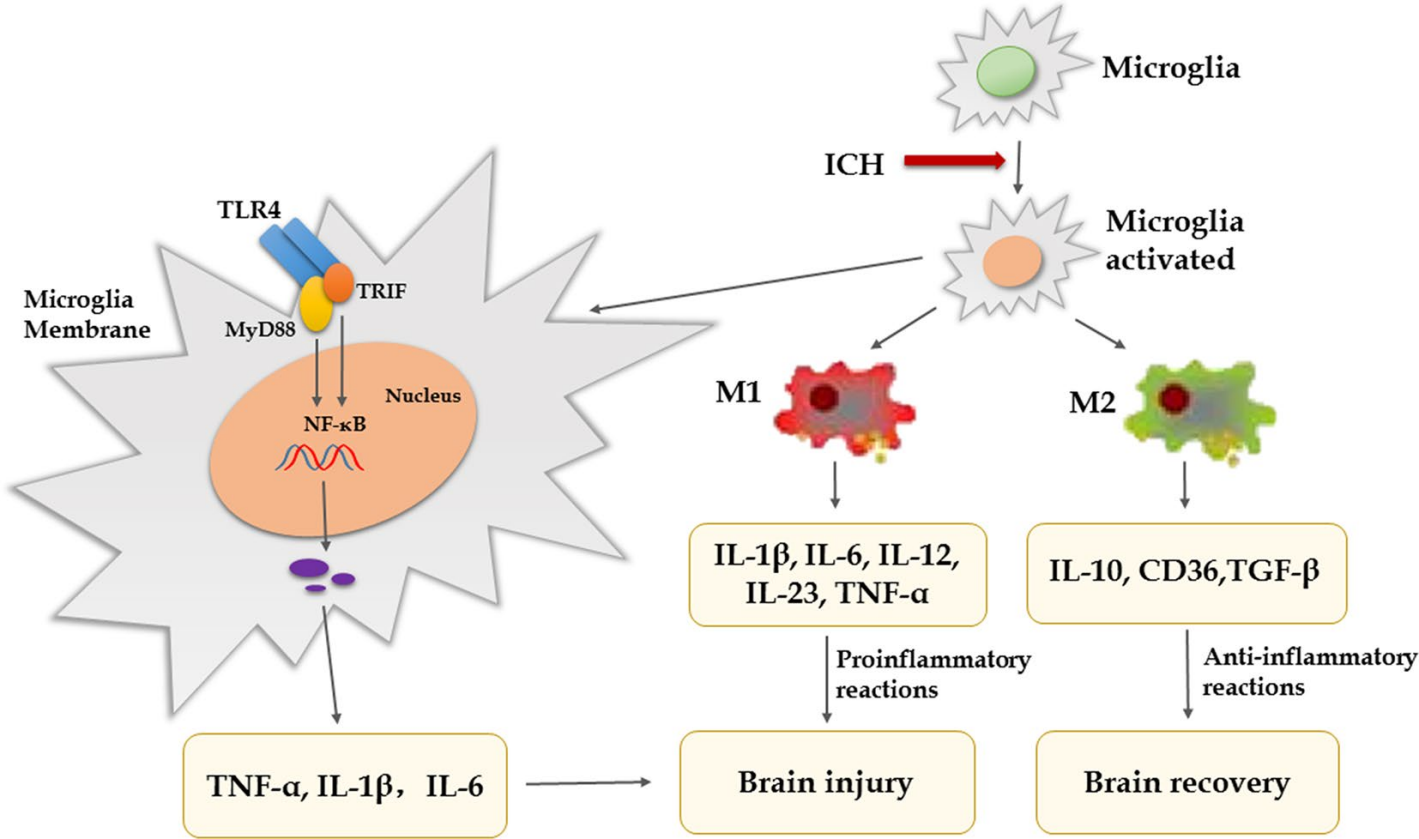
Pro- and anti-inflammatory cytokines in secondary brain injury after ICH.

Substantial evidence indicates that inflammatory mechanisms are associated with ICH-induced brain injury and microglia/macrophages activation, and polarization is thought to play vital pathophysiological roles (Wan et al., 2016; Koh et al., 2018). Under physiological conditions, microglia/macrophages monitor the surrounding microenvironment and maintain the stability of neurons, blood–brain barrier (BBB) and matrix. When cerebral hemorrhage occurs, it is activated rapidly. Excessive microglia/macrophages will release a large number of inflammatory factors and induce inflammatory waterfall reaction, which will eventually lead to pathological changes such as BBB injury, edema, cell death, and so on (Bhatia et al., 2016). In the early stage of ICH, activated microglia have been proven to produce pro-inflammatory factors and are associated with neurologic functional damage, and inhibiting microglial activation can decrease brain injury and edema (Wu et al., 2008; Scott et al., 2018). After microglia/macrophages activation, there are two types of cells, including classically activated microglia/macrophages (M1 phenotype) and alternative activated microglia/macrophages (M2 phenotype) (Xiong et al., 2016). In the early phase of ICH, microglia/macrophages can be activated by various components in the blood and readily turn into the M1 phenotype. M1 express a large number of toll like receptor 4 (TLR4) and heme oxygenase 1 (HO-1) to clear the hematoma, but they also produce proinflammatory mediators [interleukin (IL)-1β, IL-6, IL-12, IL-23, and tumor necrosis factor alpha (TNF-α)], iron content, and oxidative metabolites, which aggravate brain injury (Varnum and Ikezu, 2012; Ponomarev et al., 2013; Scott et al., 2018). M2 can improve brain recovery by secreting IL-10, CD36, and transforming growth factor-β (TGF-β) to clear cell debris and reduce inflammation, and are associated with tissue remodeling (Pan et al., 2015; Xia et al., 2015; Zheng et al., 2016; Zhang et al., 2018). Therefore, it indicates that promoting M2 phenotype and inhibiting M1 phenotype are beneficial to brain recovery after ICH. Research revealed that pinocembrin can reduce the number of M1-like microglia without affecting M2-like microglia in the surrounding area (Lan et al., 2017). A study showed that advanced glycation end products (AGEs) could cause non-specific neuroinflammation by activating the RAGE/Rho/ROCK pathway and inhibition of RAGE/ROCK not only avoids polarization of pro-inflammatory macrophages (M1) but also promoted shifting of M1 phenotype to M2 (Chen et al., 2017). Furthermore, there are several studies that show activated microglia express high levels of TLR4, which is closely related to neuroinflammation, infiltration of leukocytes, production of cytokines, and chemokines after ICH (Sansing et al., 2011; Lin et al., 2012; Gang et al., 2018). In experimental ICH, blockade of TLR4 reduced neuronal loss and edema formation and improved neurological function (Lin et al., 2012). What’s more, the TLR4 inhibitor ethyl (6R)-6-[*N*-(2-chloro-4-fluorophenyl) sulfamoyl]-cyclohex-1-ene-1-carboxylate (TAK-242) can promote hematoma resolution and attenuate neurological deficit (Fang et al., 2014). TLR4 is stimulated by exogenous or endogenous ligands through two main signaling pathways, myeloid differentiation factor 88 (MyD88)-dependent pathway and MyD88-independent pathway. One study has showed that MyD88 is the main signaling pathways of TLR4-induced inflammatory reaction (Gang et al., 2018). The intracellular domain of TLR4 binds to the carboxyl terminal of downstream MyD88 or Toll/IR-1 domain containing adaptor protein inducing interferon-beta (TRIF), activates downstream NF-κB induced kinase (NIK), and finally activates NF-κB, which promotes microglial secretion of TNF-α, IL-1β, and IL-6 (Pearson et al., 1999; Vezzani et al., 1999; Kong and Le, 2011; Gang et al., 2018). Lin et al. (2012) have confirmed that the secretion of inflammatory factors and microglia/macrophages infiltration is reduced after ICH in MyD88 gene knockout mice. TNF-α produced by microglia/macrophages plays a central role in neuronal damage after brain injury (Lambertsen et al., 2005; Rodriguez-Yanez and Castillo, 2008). The ICH-induced brain edema is significantly decreased in TNF-α knockout mice compared to wild-type mice, and another set of studies show that treatment with TNF-α antibody after ICH can inhibit microglia/macrophage activation and lead to less brain edema and better recovery of neurological function (Hua et al., 2006; Behrouz, 2016). Another study showed that although TNF-α inhibitors can reduce the degree of brain edema, inflammation, and neurologic impairment, they do not alter hematoma volume (Lei et al., 2013). IL-1β produced by microglia/macrophages is also considered a key mediator of neuronal injury; some studies show that neuroprotection is associated with downregulation of IL-1β (Wu et al., 2010; Bimpis et al., 2015). To sum up, inflammation mediated by TLR4 signaling pathway lead to brain injury after ICH, which provides us with a potential therapeutic target of ICH. We may use TLR4 antagonists and some negative regulators of TLR4 signal pathways or inhibit inflammatory factors to intervene with TLR4 downstream signal transduction, so as to treat ICH and improve the prognosis. However, it is noteworthy that microglia/macrophage-mediated phagocytosis is advantaged for tissue repair and functional recovery (Wang and Tsirka, 2005; Zhao et al., 2007). CD36 is a class II scavenger receptor of microglia/macrophages, which is closely related to phagocytosis (Flores et al., 2016). A study showed that CD36 expression was increased in perihematomal tissues in mice after ICH, while the absorption of hematoma was decreased in CD36(−/−) mice (Fang et al., 2014). Treating animals with peroxisome proliferator-activated receptor gamma (PPARγ) agonists (e.g., rosiglitazone, pioglitazone, or 15d-PGJ2) increased CD36 expression levels and enhanced hematoma resolution after ICH (Flores et al., 2016). Therefore, in addition to blocking microglia-mediated inflammatory response, promoting phagocytosis of microglia is also a therapeutic direction. However, the microglia that ingest more than two red blood cells (RBCs) lead to the release of harmful heme and iron into the extracellular matrix (Kondo et al., 1988). Prostaglandin (PG) E2-mediated inflammatory response is involved in ICH-induced secondary brain injury (Mohan et al., 2012). PGE2 has four different G-protein-coupled receptor subtypes known as EP1–EP4. Compared with the toxicity of the EP1/EP3 receptor, the EP2 receptor has neuroprotection after ICH (Mohan et al., 2012; Wu et al., 2017). Previous study found that misoprostol, an ep2/ep4 receptor agonist, can protect the brain from ICH damage (Wu et al., 2015). In addition, the synthesis of PGE2 is catalyzed by cyclooxygenase and PGE2 synthase. Celecoxib is a selective inhibitor of COX-2 that can reduce ICH-induced brain damage (Shao et al., 2019). However, excessive suppression of inflammation after ICH not only increases the risk of infection but also hinders tissue repair and hematoma clearance (Tapia-Perez et al., 2016). Therefore, regulating the balance between pro-inflammatory and anti-inflammatory responses is the pivotal point (**Figure 1**).

### Oxidative Stress

**Figure 2:**
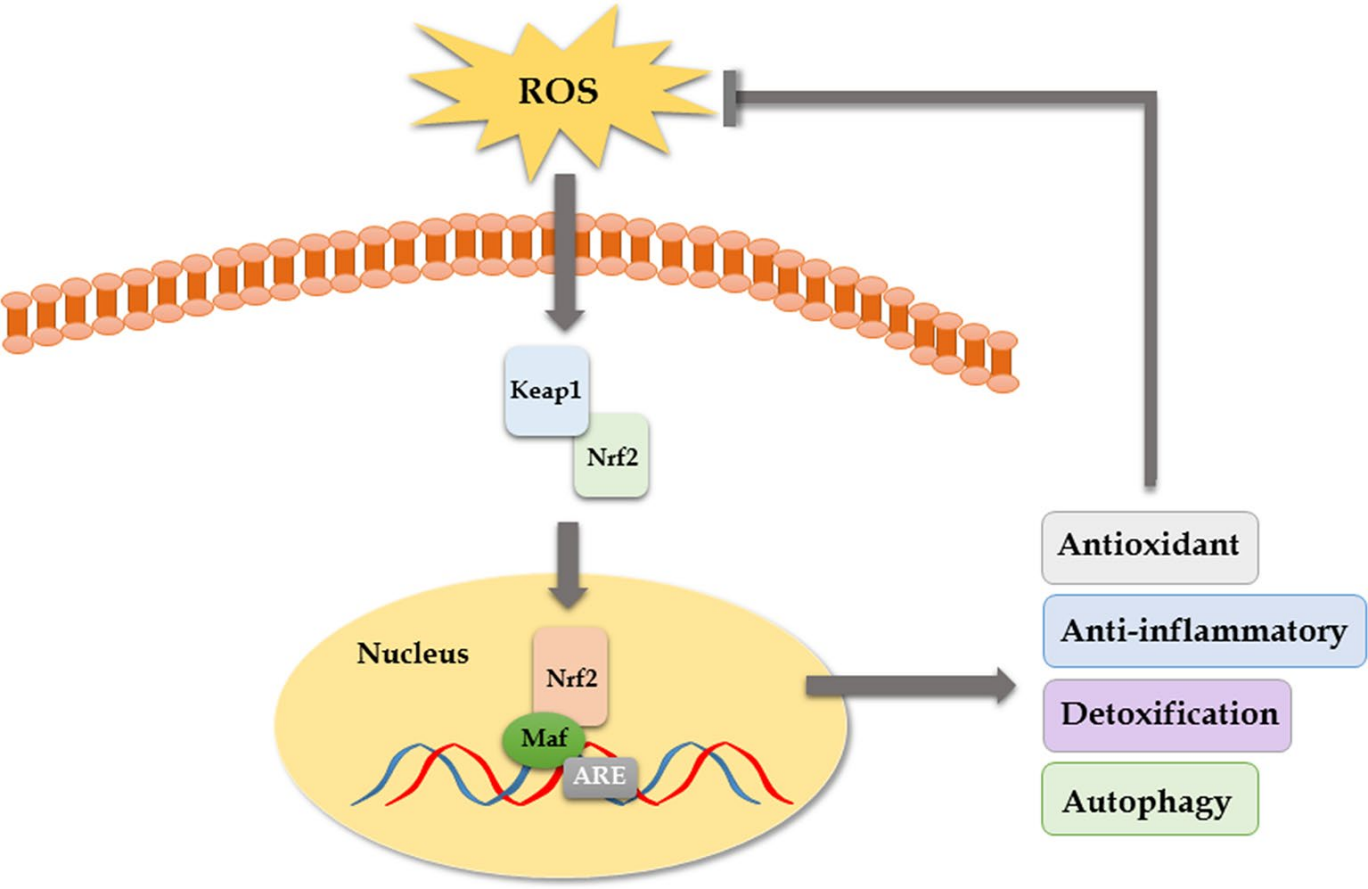
Mechanisms of erythrocyte lysates and thrombin in secondary brain injury after ICH.

OS has been increasingly recognized as a contributing factor in secondary brain injury (SBI) following ICH. OS is a condition in which there is an overproduction of free radicals, mainly reactive oxygen species (ROS), and it is involved in various important stages of pathophysiological response during ICH (Aronowski and Zhao, 2011). During physiological conditions, the organism relies on the free radical scavenging system to maintain a relative balance between the production and scavenging of free radicals. However, once the oxygen free radicals are overproduced or the clearance is weakened, it will lead to cell damage or death (Duan et al., 2016). Moreover, the central nervous system consumes more oxygen, while its endogenous antioxidant defense capacity is lower than other organs, which makes it more susceptible to OS (Liu et al., 2019). Firstly, the inflammation after ICH can produce large numbers of free radicals, which can lead to brain injury (Keep et al., 2012; Duan et al., 2016; Zheng et al., 2016). During the inflammatory response following ICH, activation of neutrophils leads to outbreak of the respiratory chain, releasing a lot of ROS and nitric oxide, and the superoxide dismutase (SOD) is consumed in large quantities to eliminate free radicals, which ultimately results in excessive lipid peroxidation (Yu et al., 2014). Excessive oxidation of lipids alters the physical properties of cellular membranes and can cause covalent modification of proteins and nucleic acids, leading to brain injury (Gaschler and Stockwell, 2017). Secondly, blood cell decomposition products such as iron ions and heme can directly lead to brain damage by producing a lot of free radicals (Wagner et al., 2003; Li et al., 2017). As a scavenger for oxygen free radicals and antioxidant, edaravone can relieve oxidative damage of neurons by scavenging free radicals and inhibiting lipid peroxidation in mice (Lu et al., 2012; Wu et al., 2014). In a published clinical trial, edaravone significantly improved the NIHSS score in patients with ICH after removal of hematoma with minimally invasive surgery (Zhao and Liu, 2014). Though its neuroprotective function has been confirmed in some preclinical and clinical studies, the effect of edaravone in ICH remains unclear because of a lack of multicenter, randomized, double-blind clinical trials (Yang et al., 2015). Some studies have shown that the nuclear transcription factor Keap1/Nrf2 pathway is the core regulatory hub of antioxidant defense system and plays an important role in the repair of SBI such as cerebral hemorrhage and ischemia (Dang et al., 2012). ROS can activate the Keap1/Nrf2 pathway to counteract oxidative damage after ICH as an adaptive response. When the Nrf2 gene was knocked out, the ICH model of mice showed increased hemorrhage volume, leukocyte infiltration, ROS production, and DNA damage. However, when Nrf2 was overexpressed, the abovementioned manifestations were significantly alleviated (Wang et al., 2007; Zhao et al., 2007). Gene silencing of Nrf2 aggravated brain edema and neuronal degeneration. Isoliquiritigenin (ILG) is a flavonoid with a chalcone structure, which can relieve early brain injury and neurological deficits by activating the Nrf2-mediated antioxidant system (Zeng et al., 2017). Moreover, it has been reported recently that nicotinamide mononucleotide treatment significantly reduced brain edema, brain cell death, OS, neuroinflammation, intercellular adhesion molecule-1 expression, microglia activation, and neutrophil infiltration in brain hemorrhagic area by promoting the activation of Nrf2 signaling pathway and inhibiting OS (Wei et al., 2017). PPARγ agonists also have been reported to function as an anti-oxidant by activating the Nrf2 pathway and increasing catalase and SOD (Zhao et al., 2015). In conclusion, antioxidant therapy is a worthy and promising ICH treatment direction (**Figure 2**).

### Cytotoxicity of Erythrocyte Lysates

**Figure 3:**
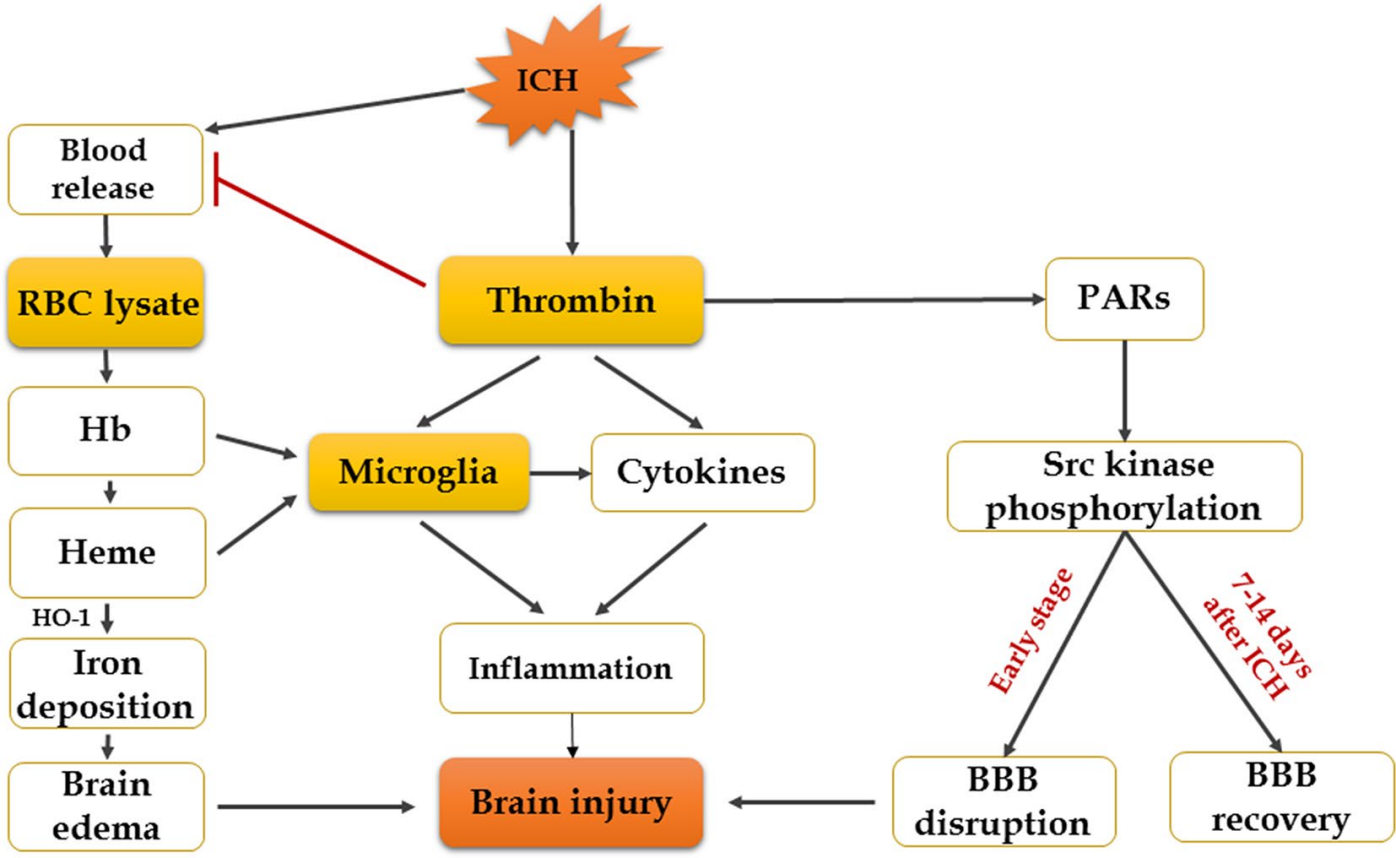
The Keap1–Nrf2–ARE pathway. Keap1 is an OS sensor and negatively regulates Nrf2. Once exposed to ROS, the activated Nrf2 translocates to the nucleus, binds to antioxidant response element (ARE), heterodimerizes with one of the small Maf (musculoaponeurotic fibrosarcoma oncogene homolog) proteins, and enhances the upregulation of cytoprotective, antioxidant, anti-inflammatory, and detoxification genes that mediate cell survival.

Increasing evidence suggests that hemoglobin and iron release from the hematoma is a major contributor to brain injury induced by ICH (Zhang et al., 2017). After cerebral hemorrhage, a lot of RBCs that contain large numbers of hemoglobin are released into the brain’s parenchyma, and RBCs will be broken down 24 h later, resulting in hemoglobin disintegrating into heme and iron (Wagner et al., 2003). Experiments have shown that infusion of dissolved RBCs into rat striatum results in increased brain water content and neurological injury (Wu et al., 2002). Infusion of hemoglobin and hemin can promote the inflammatory response of brain injury (Huang et al., 2002; Wang et al., 2014). The mechanism of brain injury produced by erythrocyte lysates is multifaceted, and researchers have found that there are four main aspects: inflammation, oxidation, nitric oxide scavenging, and edema. Firstly, overaccumulation of iron can do harm to the brain, and HO-1, the initial enzyme and rate-limiting enzyme of heme metabolism, will be expressed increasingly after ICH, which can exacerbate brain injury by promoting microglial activation and iron deposition (Lin et al., 2012; Zhang et al., 2017). However, recent studies also showed that hemin-induced HO-1 expression in perivascular cells before ICH can attenuate BBB disruption after ICH (Lu et al., 2014). Although the role of HO-1 in ICH has been extensively studied, the effect of HO-1 in ICH remains controversial. Secondly, another mechanism by which iron might cause tissue injury is the generation of free radicals. It is suggested that divalent iron ions can react with lipid to produce ROS and lipid ROS, leading to neurological damage and oxidative brain injury (Katsu et al., 2010; Li et al., 2017). Therefore, the iron chelator deferoxamine mesylate (DFX) is a promising candidate for ICH patients (Yeatts et al., 2013). DFX can cross the BBB and chelate iron ions and generate a stable complex with ferric iron; it also reduces the production of free radicals (Yeatts et al., 2013; Zeng et al., 2018). According to the mechanisms mentioned above, DFX decreases OS and neuronal death and improves functional outcome after ICH (Zhang et al., 2017). However, others claim that, although DFX can diminish total parenchymal iron levels, it does not alleviate damage or improve neurological function (Auriat et al., 2012). Thirdly, nitric oxide is depleted rapidly by hemoglobin, which produces microthrombosis in cerebral vessels in SAH and leads to brain damage (Bulters et al., 2018). Nitric oxide donors have turned out to be beneficial for ICH and SAH (Oldfield et al., 2013). Finally, there are evidences that hemoglobin and its decomposition products are main causes of edema (Wang et al., 2018). Previous study has shown that autologous blood injection will result in BBB disruption and brain edema in the rat model (Yuan et al., 2019). It has recently been demonstrated that estrogen reduces ferrous iron toxicity *in vivo* and *in vitro*, indicating that estrogen may be a potential therapeutic drug in ICH (Gu et al., 2010). Some animal experiments showed that administration of ferrostatin-1 can reduce iron deposition induced by hemoglobin and prevent neuronal death in organotypic hippocampal slice cultures (OHSCs) by inhibiting lipid ROS and COX-2 expression (Wu et al., 2011; Li et al., 2017; Zille et al., 2017). Haptoglobin–hemoglobin–CD163 acts as a main pathway in hemoglobin scavenging after ICH. A study has shown that PPAR-agonist significantly reduces hematoma volume, brain edema, and hemoglobin by promoting this signal pathway after ICH onset (Wang et al., 2018). All in all, reducing iron accumulation and erythrocyte lysate toxicity needs further study; however, continues to be an important direction in the treatment of ICH patients (**Figure 3**).

### Neurotoxicity of Thrombin

Thrombin is an essential component in the clotting cascade, and it is produced in the brain immediately after ICH induction (Zheng et al., 2016). However, thrombin can also participate in ICH-induced injury. The deleterious or protective effect of thrombin depends on its concentration (Zhu et al., 2019). It may provide neuroprotective effects against OS and ischemic injury at very low concentrations, while direct infusion into the brain of large doses of thrombin causes inflammatory cells to infiltrate the brain, proliferation of mesenchymal cells, formation of scar tissue and brain edema, and seizures (Xi et al., 2003). In the cleavage of fibrinogen to fibrin, the effects of thrombin can be mediated by non-receptors, while in the activation of p44/42 mitogen activated protein kinase, it is receptor-mediated, which contains three protease-activated receptors (PARs): PAR-1, PAR-3, and PAR-4. Protease-activated receptors, especially PAR-1, are closely related to brain damage after ICH (Guan et al., 2004; Cheng et al., 2014). Thrombin can also activate Src kinase, which might contribute to the disruption of BBB and formation of edema through PARs (Liu et al., 2010). From the above, it proves that inhibition of thrombin, PAR-1 activation, or Src kinase is a potential effective way to relieve secondary injury after ICH. In fact, a thrombin inhibitor, argatroban, has been proven to exhibit a significant neuroprotective effect and suppress the pathological progression following ICH by preventing thrombin cytotoxicity *in vitro* (Ohnishi et al., 2007). In addition, thrombin-induced injury to the BBB after ICH can be blocked by acute administration of hirudin (a direct peptide mimetic thrombin inhibitor) (Liu et al., 2010). However, thrombin inhibitors may not be an assured form of treatment, because these might affect clotting and hemostatic functions (Zheng et al., 2016). It is becoming clear that the balance between the hemostatic and pro-hemorrhagic actions of thrombin is likely dependent on multiple factors such as site (intra- or extravascular) and mode of action (activation of which type of receptor) and so on (Cheng et al., 2014). Therefore, it is critical to block the neurotoxic effects of thrombin without inhibiting its hemostasis effect. A study suggested that acute administration of the Src inhibitor PP2 can block the thrombin pathway and reduce brain edema following ICH without affecting coagulation (Liu et al., 2010; Liu and Sharp, 2011). Another set of studies showed that systemic administration of PP2 or intraventricular injection of siRNA-Fyn, a Src family kinase family member, prevented hippocampal neuronal loss and spatial memory deficits following intraventricular hemorrhage (Liu et al., 2017). Thus, the targeted use of PAR-1 or Src kinase inhibitors may possibly represent a future therapeutic method to relieve the toxic consequences of thrombin in ICH (Liu and Sharp, 2011; Gao et al., 2014; Liu et al., 2017). However, Src kinase proto-oncogene members can also promote BBB repair and brain edema resolution in the recovery stage (7–14 days) after ICH (Liu and Sharp, 2011). More studies are required to address which specific Src family members may mediate ICH-induced brain injuries.

## Conclusion

The pathophysiology mechanism of injury after ICH is very complicated, which contains OS, inflammation, nerve cell toxicity, thrombin formation, and so on. Preclinical and clinical research evidence in ICH has further revealed the progress of pathophysiology in cerebral hemorrhage, and these works have resulted in much new information about injury mechanisms and potential therapeutic targets. However, truly effective clinical treatments are very limited, mainly because the problem of transforming preclinical research into clinical application has not yet been solved. Therefore, a multi-target neuroprotective therapy will make clinically effective treatment strategies possible, but also requires further study.

## Author Contributions

ZS and ST drafted the manuscript. AS reviewed and modified the manuscript. All authors agreed on the final version.

## Funding

This work was funded by the China Postdoctoral Science Foundation (2017M612010) and the National Natural Science Foundation of China (81701144).

## Conflict of Interest Statement

The authors declare that the research was conducted in the absence of any commercial or financial relationships that could be construed as a potential conflict of interest.
